# Effect of dietary supplementation with aspirin eugenol ester on performance and ileum health in broilers under high stocking density stress conditions

**DOI:** 10.3389/fvets.2025.1638245

**Published:** 2025-07-07

**Authors:** Penghui Ma, Yi Zhang, Dongying Bai, Wenrui Zhen, Caifang Guo, Koichi Ito, Bingkun Zhang, Yanbo Ma

**Affiliations:** ^1^Department of Animal Physiology, College of Animal Science and Technology, Henan University of Science and Technology, Luoyang, China; ^2^Henan International Joint Laboratory of Animal Welfare and Health Breeding, College of Animal Science and Technology, Henan University of Science and Technology, Luoyang, China; ^3^Department of Food and Physiological Models, Graduate School of Agricultural and Life Sciences, The University of Tokyo, Ibaraki, Japan; ^4^State Key Laboratory of Animal Nutrition, Department of Animal Nutrition and Feed Science, College of Animal Science and Technology, China Agricultural University, Beijing, China; ^5^Innovative Research Team of Livestock Intelligent Breeding and Equipment, Science and Technology Innovation Center for Completed Set Equipment, Longmen Laboratory, Luoyang, China

**Keywords:** broiler, stocking density, aspirin eugenol ester, microbiology, production performance

## Abstract

This study investigated the influence of dietary aspirin eugenol ester (AEE) on growth performance, ileum antioxidant capacity, intestinal barrier function and ileum microbiota of broilers subjected to high stocking density stress (HDS). A total of 360 one-day-old Arbor Acres broilers were randomly assigned to four treatment groups: normal density (ND, 14 broilers/m^2^), normal density + AEE (NDAEE), high density (HD, 22 broilers/m^2^) and high density + AEE (HDAEE). HDS leads to a significant decrease in broiler performance while reducing the antioxidant capacity of the gut. Morphologic examination of the intestine revealed that HDS causes damage to the intestinal villi. Dietary addition of AEE significantly increased body weight gain and improved antioxidant capacity with restoration of intestinal morphology in broilers with HDS. In addition, HDS resulted in decreased gene expression of ileum tight junction proteins and significantly increased gene expression of inflammatory factors in broilers. Dietary addition of AEE effectively alleviated the decrease in tight junction protein gene expression in broilers with HDS and reduced the expression of HDS-induced inflammatory cytokines through the *COX-2-mPGES-1* signaling pathway. AEE supplementation improved microbiota diversity and increased the relative abundance of *Lactobacillus* and *Ileibacterium*. Thus, dietary AEE effectively reduced the negative effects of HDS on productivity, gut microbiota and overall health in broilers and could be a worthwhile dietary supplement for offsetting negative effects of stocking stress in broilers.

## 1 Introduction

Poultry, particularly broiler chickens, are an important source of affordable, high-quality animal protein. Due to their short growth cycle, low feed consumption and desirable meat quality, broilers play a crucial role in filling the shortage of livestock and poultry meat products (1). With declining production costs and the full recovery of broiler consumption worldwide, global broiler production is expected to reach 103.26 million tons in 2025. Driven by growing demand for broilers and in an attempt to reduce production costs, companies are increasingly adopting intensive-scale breeding methods, which have been previously shown to improve profitability (2).

In broiler farming, stocking density is typically expressed as animal weight or number of animals per unit area. In fact, broiler stocking density is strictly regulated through a comprehensive assessment of performance, animal welfare and behavioral indicators. Currently, the permitted stocking density is 16 animals/m^2^ or 39 kg/m^2^ (3–5). Usually elevated temperature in the rearing environment is considered as the main factor for poor performance under high density conditions. High temperatures can lead to adverse effects such as metabolic disorders and immunosuppression resulting in decreased production. To maximize profit, some breeding companies may attempt to increase stocking density. However, as stocking density increases, broiler growing conditions deteriorate due to factors such as rising environmental temperature, which affects their physiological condition, disturbs the balance of the internal environment, and consequently affects behavior and growth performance. This practice poses significant risks to animal health and welfare, thereby reducing economic efficiency (6–8). Compared with other stress sources, density stress is a complex, integrative stressor that originates from diverse stress sources (9, 10). For example, high stocking density stress (HDS) results in increased ambient temperatures, increased concentrations of toxic and hazardous gasses in the environment, and increased competition for food resources among broilers (11–14). With stocking density exceeding a critical level, the accumulation of toxic gasses and rise in ambient temperature disrupt the oxidative and antioxidant balance of the organism (15, 16). Subsequent accumulation of reactive oxygen species (ROS) results in oxidative stress, which affects the organism’s normal physiological and biochemical reactions (17–19). Previously, a significant association between reduced antioxidant capacity and the manifestation of various stress responses was reported. Important components of the antioxidant defense system include serum catalase (CAT), total antioxidant capacity (T-AOC), and superoxide dismutase (SOD) while malondialdehyde (MDA) is a marker of oxidative damage in the body (20, 21). Previous studies indicated that increased stocking density stress negatively affected various serum parameters, leading to increased oxidative damage in broilers, which in turn affects broiler performance (22, 23).

The intestine represents a crucial defense organ against pathogenic microorganisms and toxins (24, 25). HDS leads to a reduction in the height of intestinal villi (VH) and increases crypt depth (CD), which compromises the intestinal physical barrier and impairs nutrient absorption (26, 27). HDS was previously shown to affect intestinal integrity at multiple levels. HDS correlates with reduced levels of the intestinal tight junction proteins occludin, claudin, and *ZO-1* (28, 29), which are markers of changed intestinal morphology (30, 31). It has been shown that HDS leads to reduced broiler performance, impaired fluff morphology, and disruption of the microbiota in broilers (31). Also, HDS triggers an inflammatory response in the intestine characterized by enhanced levels of the pro-inflammatory factors *IL-1β*, *IL-10*, *TNF-α* (29, 31, 32). This intestinal inflammatory response results in Cyclooxygenase-2 (*COX-2*)-mediated prostaglandin G2 production from arachidonic acid, which is subsequently converted to prostaglandin H2 and E2 (*PGE2*) by prostaglandin E synthase (*PGES*) (33). Ultimately, this cascade results in intestinal barrier disruption, the development of inflammatory bowel disorders, and inflammatory responses in intestinal epithelial cells (34). Interestingly, inflammatory stimuli upregulate microsomal prostaglandin E synthase-1 (*mPGES-1*), which exhibits expression patterns similar to *COX-2* and acts at the final stage of *PGE2* synthesis, controlling its production rate (35). HDS has been proven to damage the intestinal barrier and increase intestinal permeability, leading to significant changes in the composition of the cecum microbiota, specifically an increase in the relative abundance of the genus *Blautia* and decreases in *Lactobacillus* and *Bifidobacterium*. These changes in the gut microbiota affect digestive efficiency and nutrient absorption, negatively impacting poultry production (31). It is evident that HDS has evolved into a significant threat not only to animal welfare but also to the economic viability of contemporary poultry farming enterprises. Consequently, there is an imperative for the expeditious development of a novel anti-stress strategy.

Considering the significant impact of HDS on the economic performance of poultry farms, it is common practice to use food supplements to mitigate HDS-mediated factors (36). Aspirin eugenol ester (AEE) is a novel compound formed by the condensation of aspirin and eugenol through ester bonding with similar anti-inflammatory, antimicrobial, and antioxidant effects of the two bioactive compounds. However, it lacks aspirin-mediated stomach irritation (37, 38); while enhancing eugenol stability (39). Interestingly, AEE has a favorable safety profile as it has a lower toxicity and longer duration of action of its two building compounds alone (40). Meanwhile, dose residue analysis in rats experiments showed that the organism was able to metabolize and excrete AEE within a short period of time (41). Importantly, AEE significantly reduced *COX-1* and *COX-2* as well as *IL-1β*, *IL-6*, and *IL-10* expression in mice. Furthermore, studies of rat cecum microbiome and metabolism in hyperlipidemia rats evidenced an AEE-mediated restoration toward normal nutrient absorption and metabolic functions through intestinal microbiota composition alteration (41, 42). Of note, HDS broilers showed an AEE-mediated restoration of caecum microbiota diversity with increased abundance of the beneficial bacterium *Lactobacillus* (23).

As most previous studies on AEE focused on *in vitro* cell culture and rat models, further research is needed to directly evaluate the effects of AEE on ileum health (morphology, barrier, inflammation, microbiota) in HDS broilers and elucidate its anti-inflammatory mechanisms. Concurrently, AEE has been demonstrated to possess anti-inflammatory efficacy in murine models; however, the precise mechanisms underlying its anti-inflammatory effects require further elucidation. Therefore, the present study aimed to investigate the effects of AEE on growth performance, ileum antioxidant properties, ileum physical barrier morphology, ileum inflammatory factor gene expression, and ileum microbiota in HDS broilers, and to analyze the underlying mechanisms using a HDS model.

## 2 Materials and methods

Institutional Review Board Statement: Animal experiments were approved by the Animal Care and Use Committee of Henan University of Science and Technology (DWFL36891-2023) on 9 October 2023.

### 2.1 Experimental design, animals and feeding

A total of 360 healthy, similar weight, 1-day-old male Arbor Acres broilers (Henan Quanda Poultry Breeding Company Co., Ltd., Henan, China) were randomized into four treatment groups: normal density groups (ND), normal density + AEE groups (NDAEE), high density groups (HD), and high density + AEE groups (HDAEE), each with 10 replicates. Ten replicate groups were set up for each of the above four treatment groups, totaling 40 cages. The ND group was housed at a density of 7 animals/cage and the HD group was housed at a density of 11 animals/cage. The dimensions of the rearing cages used in the experiment were 0.7 m long, 0.7 m wide and 0.5 m high. Throughout the experimental cycle, broilers in the ND group were kept in an average space of 0.07 m^2^/broiler, and broilers in the HD group were kept in an average space of 0.045 m^2^/broiler. The cages were fitted with easily movable nylon mesh barriers to allow us to maintain the high and low stocking densities by reducing the available cage space after each sampling (14, 17). The ND and HD groups received a basal diet, while the NDAEE and HDAEE groups received the basal diet supplemented with 0.01% AEE (Lanzhou Institute of Animal Science and Veterinary Medicine, Chinese Academy of Agricultural Sciences, >99.5% purity) (23). The basic feed ratios are listed in Table 1 (the dietary ratios and nutritional levels of broiler chickens meet the standards of GB/T 5916–2020 Compatible Feeds for Egg-laying Chickens and Broiler Chickens). AEE is a colorless and odorless crystal. After grinding, the AEE powder was thoroughly mixed with crushed feed at the specified ratio and finally processed into pellets for broiler feeding using a pelletizer. A gradient concentration pre-test was established in order to screen the optimal concentration of AEE. Following analysis of production performance, inflammatory response and immune organ index, it was determined that the optimal concentration of AEE in broiler diets was 0.01% (23). Before starting the experiment, the chicken coop and equipment were thoroughly cleaned and sterilized according to established protocols. The house was equipped with three-layer stacked cages, water pipes, and troughs to allow free feeding and drinking. The experiment was divided into two phases: 1–21 days and 22–42 days. Broilers were exposed to continuous light for the first 3 days, after which light exposure was gradually reduced, eventually reaching 6 h of darkness per day. Throughout the experiment, the broilers were vaccinated, and the rearing environment was regularly disinfected and cleaned to maintain health. The broiler vaccination program was as follows: 1 day old a subcutaneous injection of Marek’s vaccine, 7 days old Newcastle Disease IV a pass branch 120 diphtheria vaccine drops nose and eyes, 14 days old infectious bursal vaccine drops nose or eyes, 21 days old infectious bursal vaccine second drinking water immunization, 28 days old Newcastle Disease IV a pass branch 120 diphtheria vaccine second drinking water immunization.

**Table 1:** Basic diet formula and nutrient levels.

Items	1-21d	22-42d
Ingredient composition, %		
Corn	62.45	66.56
Soybean meal (43%CP)	25.61	25.25
Soybean oil	1.42	2.15
Flours	2.83	0
Talcum powder	4.05	2.97
Calcium biphosphate	1.54	1.11
Sodium chloride NaCl	0.32	0.32
Choline chloride (Ch3Cl) (50%)	0.14	0
Vitamins, minerals premix^a^	1.64	1.64
Total	100.00	100.00
Nutrient content^b^		
Metabolizable energy (MJ/kg)	12.52	13.23
Crude protein, %	20.21	18.66
Crude fiber, %	3.44	4.86
Calcium, %	1.66	1.51
Total phosphorus, %	0.67	0.58
Lysine, %	1.35	1.17
Threonine, %	0.78	0.71
Methionine + Cystine, %	1.47	1.22

a Vitamin-mineral premix was provided per kg of diet: vitamin A (vincristine acetate), 8,000 IU; vitamin D3 (cholecalciferol), 700 IU; vitamin E (DL-a-tocopheryl acetate), 18 IU; vitamin K3 (menaquinone sodium bisulfate), 2.1 mg; vitamin B1, 2 mg; vitamin B2, 6 mg; vitamin B6, 2.1 mg; Vitamin B12 (cyanocobalamin), 0.020 mg; biotin, 0.0325 mg; niacin, 20 mg, folic acid, 0.6 mg; pantothenic acid, 10 mg; biotin, 0.4 mg, manganese, 80 mg. Copper, 10 mg, zinc, 60 mg, ferrous, 40 mg, iodine, 0.5 mg, selenium, 0.2 mg.

b Nutrient levels are measured, except for metabolizable energy, which is calculated.

### 2.2 Recording of production performance and samples collection

During the experimental period, broilers were subjected to an 8-h fasting period at 21 and 42 days of age. Following fasting, body weight and feed consumption of the broilers were recorded and the average daily gain (ADG), average daily feed consumption (ADFI) and feed conversion ratio (FCR) were calculated. At 21, 28, 35, and 42 days of age, six replicates were randomly selected from each treatment group, and a single broiler was randomly chosen from each replicate. The broilers were euthanised by inhalation of carbon dioxide, after which ileum samples were isolated and collected. One cm long sections of intestine were collected and fixed in 4% paraformaldehyde for subsequent embedding and sectioning. The other parts were immediately frozen with liquid nitrogen and stored at −80°C for later measurement of antioxidant and tight junction protein levels. Lastly, the contents of the ileum were removed and placed in an enzyme-free tube, quickly frozen in liquid nitrogen, and stored at −80°C for subsequent 16S rRNA detection.

### 2.3 Intestinal tissue morphology

Ileum tissue samples were fixed in 4% paraformaldehyde for 24 h, dehydrated with an ethanol solution gradient, and treated with xylene solution. The hyalinized tissue was embedded in paraffin wax, cooled, and cut into 4–6 μm thick sections. Sections were deparaffinized, dehydrated, and stained with xylene solution as previously described (43). The stained sections were visualized with a scanner to evaluate intestinal morphology and anatomical measurements. Ten well-aligned, intact villi were selected for measurement using Case Viewer software (version 2.0). The villus height (defined as the distance from the tip of the villi to the opening of the villus crypt) and the crypt depth (the distance from the base of the intestinal gland to the opening of the crypt) as well as the ratio of the villus heights were measured and the depth of the crypt was calculated. These measurements were used to assess changes in intestinal morphology.

### 2.4 Determination of intestinal antioxidant properties

Approximately 0.1 g of sample was collected and homogenized at a tissue-to-saline ratio of 1:9, ground into a homogenate, and set aside for analysis. T-AOC was determined by the ABTS method, MDA by the thiobarbituric acid method, CAT by the colorimetric method, and SOD by the WT-1 method according to the instructions of the antioxidant kits (Nanjing Jiancheng Bioengineering Co., T-AOC: A015-2-1, MDA: A003-1, CAT: A007-1-1, SOD: A001-3, Nanjing, China).

### 2.5 Extraction of total intestinal RNA and gene expression analysis

Extraction of total intestinal RNA was performed under sterile and enzyme-free conditions at low temperature. Ileum tissue samples were thawed on ice, and 50–100 mg of samples were transferred to sterile and enzyme-free 1.5 mL Eppendorf centrifuge tubes. Total RNA was extracted by lysing the tissue with TRIzol reagent (Invitrogen Inc., Carlsbad, CA, United States), and contaminants were further removed by washing with chloroform, isopropanol, and anhydrous ethanol. RNA purity was analyzed using a Nanodrop 2000 (Thermo Scientific, Ottawa, Canada), with a purity threshold of A260/A280 ratio >1.9. The concentrations were determined from absorption measurements and the samples were stored at −80°C. Total RNA contained in the tissue samples was reverse transcribed into cDNA according to the instructions of the reverse transcription kit (Acres Bioengineering Co., Ltd., Hunan, China). The SYBR Green PCR kit was used on a CFX Connect Real-Time PCR Detection System with the following reaction conditions: initial denaturation at 95°C for 5 min followed by 40 cycles of denaturation at 95°C for 15 s and annealing at 60°C for 30 s. Gene expression results were analyzed and compared using the 2^−ΔΔCT^ method. Referring to the sequences already published by NCBI, the primer sequences of the genes were designed using Primer 3.0 and synthesized by Shanghai Shenggong Bioengineering Co. The primers used in this study are listed in Table 2. GAPDH was used as an internal reference gene.

**Table 2:** Primer sequence.

Gene^a^	Primer sequence	Length (nt)	GenBank number
OCLN	F^b^: ACGGCAGCACCTACCTCAA R^c^: GGGCGAAGAAGCAGATGAG	123	XM_025144247.2
CLDN1	F: AAGTGCATGGAGGATGACCA R: GCCACTCTGTTGCCATACCA	100	NM_001013611.2
CLDN2	F: CCTACATTGGTTCAAGCATCGTG R: GATGTCGGGAGGCAGGTTGA	131	NM_001277622.1
ZO-1	F: CTTCAGGTGTTTCTCTTCCTCCTC R: CTGTGGTTTCA TGGCTGGATC	144	XM_021098886
ZO-2	F: CACCACCACCTGTTTCTGTG R: TTCACTCCCTTCCTCTTCCA	119	NC_052572.1
TNF-α	F: GAGCGTTGACTTGGCTGTC R: AAGCAACAACCAGCTA TGCAC	176	NM_214022.1
IL-1β	F: ACTGGGCA TCAAGGGCTA R: GGTAGAAGA TGAAGCGGGTC	154	NM_214005.1
IL-10	F: AGAAATCCCTCCTCGCCAAT R: AAATAGCGAACGGCCCTCA	121	NM_001004414.2
IL-6	F: GCTGCGCTTCTACACAGA R: TCCCGTTCTCA TCCA TCTTCTC	203	NM_204628.1
COX-2	F: CCGAATCGCAGCTGAATTCA R: GAAAGGCCATGTTCCAGCAT	116	NM_001277664.2
mPGES-1	F: AGGCTCAGGAAGAAGGCATT R: CACAGCTCCAAGGAAGAGGA	153	NM_001194983.1
EP2	F: CAGCAGAGGAGGTGGCAGAG R: ACAGACGCAGACACGCAGAG	112	NC_052536.1
EP3	F: CTGTGCTGCTTTGCTTTCTTTCC R: CCTGCTTATCACTGTCTCCATCTG	120	NC_052539.1
EP4	F: AGAGGAATACCAGAGTGCCAGAC R: AGTAGGAGCCGCAGGTTCAAG	147	NC_052572.1
GAPDH	F: TGCTGCCCAGAACATCATCC R: ACGGCAGGTCAGGTCAACAA	142	NM_204305

a OCLN, occludin; CLDN1, Claudin-1; CLDN2, Claudin-2; ZO-1, Zona Occludens-1; ZO-2, Zonula Occludens-2; TNF-α, tumor necrosis factor-α; IL-1β, Interleukin-1 beta; IL-10, interleukin-10; IL-6, interleukin-6; COX-2, cyclooxygenase-2; mPGES-1, microsomal prostaglandin E2 synthase-1; EP2, G protein-coupled plasma membrane receptor 2; EP3, G protein-coupled plasma membrane receptor 3; EP4, G protein-coupled plasma membrane receptor 4; GAPDH, glyceraldehyde-3-phosphate dehydrogenase.

b F: forward primer.

c R: reverse primer.

### 2.6 Microbiological sequencing of 16S rRNA from ileum contents

The ileum contents were sent to Shanghai Paisano Bioengineering Co. for 16S rRNA gene sequencing. Nucleic acids were extracted from the pretreated samples using the OMEGA Soil DNA Kit (D5635-02) (Omega Bio-Tek, Norcross, GA, United States). The extracted DNA was subjected to 0.8% agarose gel electrophoresis to determine the molecular size and the DNA was quantified using Nanodrop. The highly variable V3V4 region of the bacterial 16S rRNA gene—approximately 486 bp long—was used for sequencing in the Bacterial Intestinal Contents Project. PCR amplification was performed using universal primers 338 F: ACTCCTACGGGGAG GCAGCA and 806 R: GGACTACHVGGGGTWTCTAAT. The PCR-amplified DNA fragments were collected and stored at 12°C. Libraries were prepared using the Illumina TruSeq Nano DNA LT Library Prep Kit and two-part 2 × 250-bp sequencing was performed on an Illumina NovaSeq instrument using the NovaSeq 6000 SP Reagent Kit (500 cycles). The data was quality controlled and analyzed. The biological information of the microbiome was analyzed using QIIME2 version 2019.4, with the process modified and refined according to the official tutorial.^1^ Raw sequence data was processed using the Demux plugin for decoding, the Cutadapt plugin for primer excision, and the DADA2 plugin for data processing such as quality filtering, noise reduction, splicing, and chimera removal of the sequences. The Greengenes 2 database was used to compare and annotate the ASV feature sequences with the reference sequences in the database. Alpha diversity and Beta diversity were analyzed using Qiime2 software scripted in Python (version 3.9). Alpha diversity indices included Shannon, Chao1 and Simpson indices. The Shannon index measured the richness and evenness of species diversity within the sample. Chao1 index estimated the species richness within the sample, while Simpson index reflected the evenness of species distribution within the sample. Beta diversity was analyzed by principal coordinate analysis (PCoA) through Jaccard distance matrix. To compare Alpha diversity indices between groups, we used one-way analysis of variance (ANOVA). For Beta diversity, we performed a permutation multivariate analysis of variance (PERMANOVA) via the scikit-bio package for python. All statistical analyses were performed under R version 4.3.3. The linear discriminant analysis effect size (LEfSe) method was used to detect taxonomic units that had many differences between groups with thresholds of *p* < 0.05 and LDA = 2. Data were analyzed using the online platform Personalbio GenesCloud.^2^ The sample FASTQ information was submitted to the NCBI (Accession No: PRJNA1193887).

### 2.7 Statistical analysis

Initial data processing was conducted using Microsoft Excel 2021. The sample’s basic characteristics were described through the calculation of mean and standard deviation. Subsequently, one-way analysis of variance (ANOVA) was conducted using SPSS 22.0 (IBM, Chicago, IL, United States), followed by Duncan’s multiple comparisons test. Graphs were plotted using GraphPad Prism 9.0 (GraphPad Software, San Diego, CA, United States) with *p* < 0.05 considered as statistically significant.

## 3 Results

### 3.1 Effect of AEE on growth performance in HDS broilers

First, the performance of broiler production in different treatment groups was analyzed. The results of production performance between treatment groups are shown in Table 3. From day 1 to 21, no significant differences in ADFI, ADG, and FCR were observed between the treatment groups. From days 21 to 42, compared to the ND group, animals in the HD group had significantly reduced ADFI and ADG as well as significantly increased FCR (*p* < 0.05). From days 21 to 42, compared to the HD group, animals in the HDAEE group had similar ADFI, significantly increased ADG, and significantly decreased FCR (*p* < 0.05). It is noteworthy that dietary supplementation with AEE had no significant effect on the performance of the ND group; however, the addition of AEE to the diet significantly increased ADG and FCR in HDS broilers (*p* < 0.05).

**Table 3:** Effect of AEE on growth performance of HDS broilers.

Items	ND	HD	NDAEE	HDAEE	SEM	*p* value
1–21d						
ADFI (g)^1^	53.66	54.32	54.04	54.76	0.632	0.41
ADG (g)^2^	45.45	44.10	44.80	45.07	0.494	0.46
FCR^3^	1.20	1.22	1.21	1.20	0.013	0.77
22–42d						
ADFI (g)	153.86^a^	136.79^b^	157.21^a^	141.03^b^	2.951	0.02
ADG (g)	98.58^a^	73.48^c^	98.86^a^	80.23^b^	2.549	0.02
FCR	1.58^c^	1.86^a^	1.57^c^	1.74^b^	0.035	0.03

1 Average daily feed intake (ADFI).

2 Average daily gain (ADG).

3 Feed conversion ratio (FCR, feed: gain, g:g).

Means lacking a common superscript in a row are significantly different (*n* = 10, *p* < 0.05).

### 3.2 Effect of AEE on intestinal antioxidant properties in HDS broilers

Next, the antioxidant properties of the ileum in broilers exposed to different densities was analyzed. The results of antioxidant indexes detected between treatment groups are shown in Table 4. At 21 days of age, no significant difference in T-AOC between treatment groups was observed. From days 28 to 42, compared to the ND group, animals in the HD group had significantly lower T-AOC (*p* < 0.05). Compared to the HD group, animals in the HDAEE group had significantly higher T-AOC levels (*p* < 0.05). At 21 days of age, no significant difference in SOD activity was observed. At 28 days, SOD activity was significantly higher in NDAEE compared to HD (*p* < 0.05) while no significant difference between the HD and ND groups was observed. On days 35 and 42, animals in the HD group had significantly lower SOD activity compared to the ND group (*p* < 0.05). Compared to the HD group, animals in the HDAEE group had significantly higher SOD activity (*p* < 0.05). On 21 days of age, no significant difference in MDA content between treatment groups was observed. From days 28 to 42, MDA content was significantly increased in the HD group compared to the ND group (*p* < 0.05). Compared to the HD group, animals in the HDAEE group had significantly lower MDA content (*p* < 0.05). At 21 days of age, no significant difference in CAT content was observed. From days 28 to 42, animals in the HD group had significantly lower CAT levels compared to the ND group (*p* < 0.05). Compared to the HD group, animals in the HDAEE group had a significantly higher CAT content (*p* < 0.05).

**Table 4:** Effect of AEE on antioxidant properties of the ileum in HDS broilers.

Items	ND	HD	NDAEE	HDAEE	SEM	*p* value
T-AOC (mmol/g)^1^						
21d	0.21	0.20	0.20	0.20	0.009	0.38
28d	0.19^a^	0.16^b^	0.20^a^	0.19^a^	0.011	<0.01
35d	0.19^b^	0.16^c^	0.20^a^	0.19^b^	0.007	<0.01
42d	0.17^a^	0.14^c^	0.16^b^	0.17^a^	0.006	<0.01
SOD (U/mgprot)^2^						
21d	23.14	22.7	23.12	23.55	0.540	0.49
28d	21.45^ab^	20.09^b^	22.34^a^	21.19^ab^	0.987	0.03
35d	25.43^a^	20.28^b^	25.97^a^	25.63^a^	1.455	0.02
42d	24.77^a^	20.42^b^	24.01^a^	23.74^a^	1.075	<0.01
MDA (nmol/mgprot)^3^						
21d	0.22	0.20	0.21	0.19	0.022	0.51
28d	0.27^b^	0.34^a^	0.25^b^	0.29^b^	0.025	0.04
35d	0.25^b^	0.31^a^	0.26^b^	0.24^b^	0.015	<0.01
42d	0.19^b^	0.22^a^	0.17^c^	0.18^bc^	0.010	0.02
CAT (U/mgprot)^4^						
21d	22.05	22.01	22.34	23.67	1.130	0.42
28d	22.81^a^	18.34^b^	22.07^a^	22.44^a^	0.465	<0.01
35d	21.40^a^	19.30^b^	21.10^a^	21.49^a^	0.804	0.04
42d	21.76^a^	18.05^c^	21.75^a^	20.11^b^	0.485	0.02

1 Total antioxidant capacity (T-AOC).

2 Superoxide dismutase (SOD).

3 Malondialdehyde (MDA).

4 Catalase (CAT).

Means lacking a common superscript in a row are significantly different (*n* = 6, *p* < 0.05).

### 3.3 Effect of AEE on ileum morphology in HDS broilers

**Figure 1:**
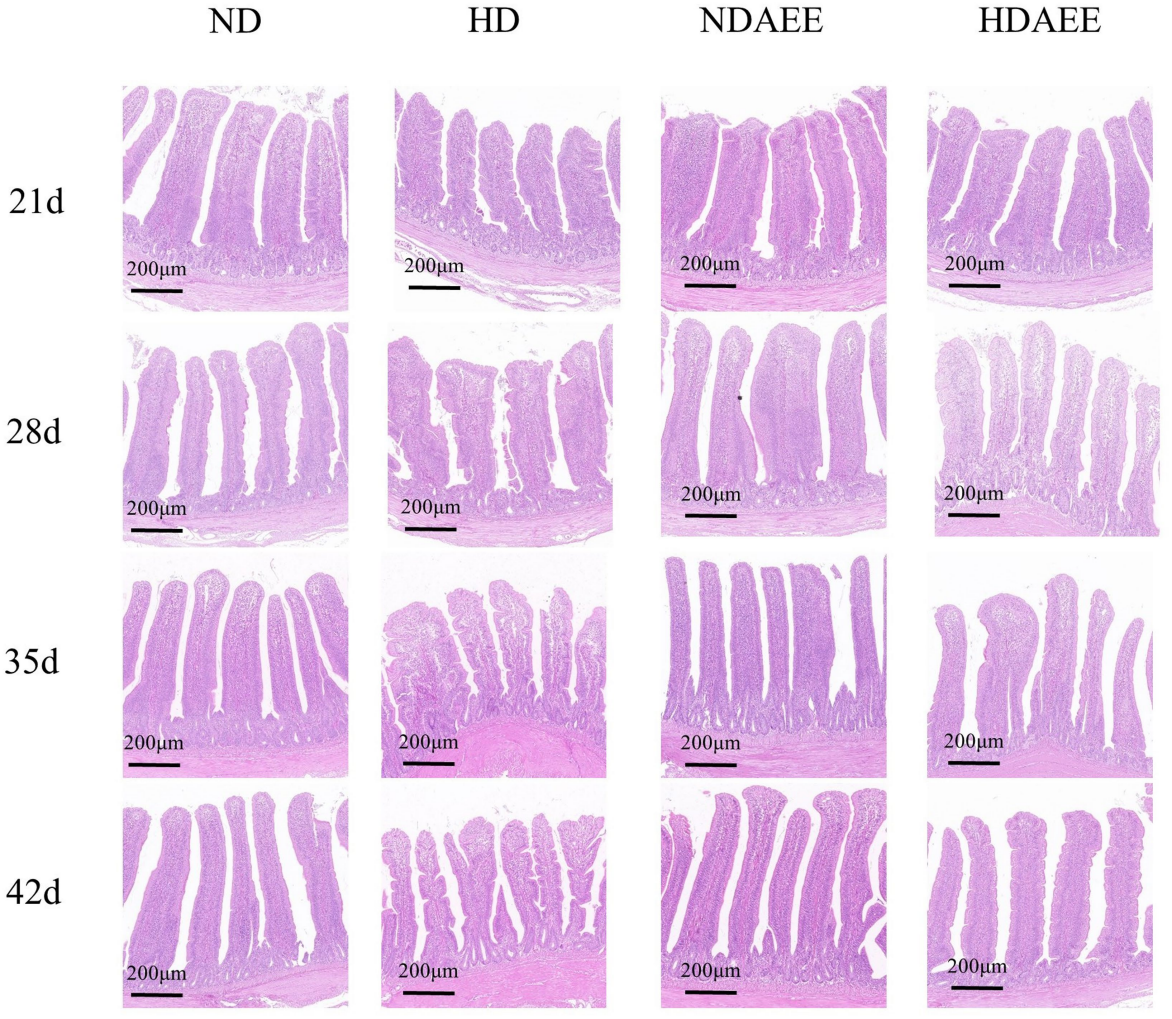
Effect of AEE on ileum Morphological structure in HDS broilers. Scale bar = 200 μm.

Then, the influence of AEE on the ileum morphology of HD broilers was analyzed. Among them, the detection results of CD with VH and V/C are shown in Table 5. At 21 to 42 days of age, animals in the HD group had significantly reduced VH compared to the ND group. Compared to the HD group, animals in the HDAEE group had significantly increased VH (*p* < 0.05). At 21 days of age, no significant difference in CD between treatment groups was observed. Compared to the ND group, animals in the HD group had significantly higher CD between 28 and 42 days. In contrast, the HDAEE group showed a significant decrease in CD compared to the HD group (*p* < 0.05). Compared to the ND group, animals in the HD group had significantly lower V/C ratio. Compared to the HD group, animals in the HDAEE group had a significantly higher V/C ratio (*p* < 0.05). The results showed that the HD group showed a decrease in villus height and accompanied by signs of villus damage compared to the ND group, while the HDAEE group was able to mitigate the damage to the intestinal morphology to a certain extent compared to the HD group (Figure 1).

**Table 5:** Effect of AEE on ileum intestinal morphology in HDS broilers.

Items	ND	HD	NDAEE	HDAEE	SEM	*p* value
VH (μm)^1^						
21d	490.42^b^	455.76^c^	583.93^a^	495.66^b^	6.440	0.03
28d	756.70^b^	662.45^c^	854.63^a^	750.34^b^	6.321	<0.01
35d	861.66^b^	703.19^d^	932.48^a^	780.46^c^	7.705	<0.01
42d	921.68^b^	833.85^c^	1051.88^a^	847.17^c^	8.950	0.04
CD (μm)^2^						
21d	60.77	61.37	59.59	60.17	0.922	0.26
28d	75.64^c^	84.88^a^	77.15^c^	79.90^b^	1.274	<0.01
35d	91.93^b^	96.38^a^	85.53^c^	91.76^b^	1.210	0.03
42d	101.87^c^	113.14^a^	102.64^c^	108.27^b^	2.234	0.03
V/C^3^						
21d	8.07^b^	7.43^c^	9.81^a^	8.24^b^	0.152	0.03
28d	10.00^b^	7.81^d^	11.10^a^	9.40^c^	0.154	0.04
35d	9.373^b^	7.31^d^	10.91^a^	8.51^c^	0.151	0.04
42d	9.05^b^	7.40^d^	10.25^a^	7.83^c^	0.157	<0.01

1 Average villus height in ileum intestine.

2 Crypt depth of ileum intestine.

3 V/C, ratio of villus height to crypt depth in ileum intestine.

Means lacking a common superscript in a row are significantly different (*n* = 6, *p* < 0.05).

### 3.4 Effect of AEE on expression of tight junction protein gene in ileum of HDS broilers

Then, the influence of AEE on the expression of ileum tight junction markers in HD broilers was analyzed. The test results are shown in Table 6. On days 21 and 28, compared to the ND group, animals in the HD group had significantly lower *occludin* gene levels (*p* < 0.05). Compared to the HD group, animals in the HDAEE group had significantly higher *occludin* gene levels (*p* < 0.05). There was no significant difference in *occludin* expression levels between the HD and ND groups at 35 and 42 days. On days 21 and 28, compared to the ND group, animals in the HD group had significantly lower *claudin-1* gene expression (*p* < 0.05). Compared to the HD group, animals in the HDAEE group showed significantly higher *claudin-1* expression (*p* < 0.05). On days 35 and 42, *claudin-1* levels were similar in all treatment groups. At 21 days of age, no significant difference in *claudin-2* expression between the ND and HD groups. Compared to the ND group, animals in the HD group had significantly lower *claudin-2* expression at 28, 35, and 42 days of age. Compared to the HD group, animals in the HDAEE group had significantly higher *claudin-2* expression levels (*p* < 0.05). On days 21, 28, and 35, compared to the ND group, animals in the HD group showed significantly lower *ZO-1* expression levels (*p* < 0.05). Compared to the HD group, animals in the HDAEE group had significantly higher *ZO-1* levels (*p* < 0.05). At 42 days of age, *ZO-1* expression levels were similar in all treatment groups. From days 21 to 42, compared to the ND group, animals in the HD group had significantly lower *ZO-2* expression (*p* < 0.05). Compared to the HD group, animals in the HDAEE group showed significantly higher *ZO-2* expression (*p* < 0.05).

**Table 6:** Effect of AEE on the relative gene expression of ileum tight junction protein in HDS broilers.

Items	ND	HD	NDAEE	HDAEE	SEM	*p* value
Occludin						
21d	1.00^a^	0.82^b^	1.05^a^	1.00^a^	0.051	<0.01
28d	1.00^a^	0.79^b^	1.02^a^	1.03^a^	0.078	0.01
35d	1.00^ab^	0.94^b^	1.13^a^	0.96^b^	0.071	0.04
42d	1.00	0.99	0.99	0.98	0.073	0.46
Claudin-1						
21d	1.00^b^	0.89^c^	1.21^a^	1.02^b^	0.052	<0.01
28d	1.00^a^	0.64^b^	1.08^a^	1.04^a^	0.073	0.02
35d	1.00	0.88	1.08	1.08	0.049	0.19
42d	1.00	0.94	1.08	1.06	0.093	0.37
Claudin-2						
21d	1.00^b^	1.08^b^	1.24^a^	1.17^ab^	0.071	0.04
28d	1.00^b^	0.70^c^	1.34^a^	1.01^b^	0.094	<0.01
35d	1.00^a^	0.81^b^	1.01^a^	0.99^ab^	0.082	0.03
42d	1.00^a^	0.90^b^	1.13^a^	1.07^a^	0.072	<0.01
ZO-1						
21d	1.00^a^	0.86^b^	1.04^a^	0.99^a^	0.053	0.01
28d	1.00^a^	0.83^b^	1.11^a^	0.98^a^	0.067	<0.01
35d	1.00^a^	0.86^b^	1.07^a^	1.08^a^	0.083	0.02
42d	1.00	1.06	1.09	1.05	0.092	0.14
ZO-2						
21d	1.00^b^	0.93^c^	1.22^a^	1.07^b^	0.081	0.01
28d	1.00^b^	0.82^c^	1.19^a^	1.04^b^	0.075	<0.01
35d	1.00^b^	0.88^c^	1.01^a^	1.01^b^	0.086	0.04
42d	1.00^a^	0.84^b^	1.01^a^	0.99^a^	0.084	0.03

Means lacking a common superscript in a row are significantly different (*n* = 6, *p* < 0.05).

### 3.5 Effects of AEE on ileum inflammatory gene expression in HDS broilers

Then, the influence of AEE on the expression of ileum inflammatory factors in HD broilers was analyzed. The results of inflammatory factor gene expression are shown in Table 7. On days 21, 28, 35, and 42, the expression levels of *IL-1β*, *IL-10*, and *TNF-α* were significantly increased in the HD group compared to the ND group, while animals in the HDAEE group had a significantly lower *IL-1β*, *IL-10*, and *TNF-α* levels compared to the HD group (*p* < 0.05). On day 21, no significant difference in *IL-6*, *COX-2*, and *mPGES-1* expression was observed between the treatment groups. On days 28, 35 and 42, the expression levels of *IL-6*, *COX-2*, and *mPGES-1* were significantly increased in the HD group compared to the ND group, while animals in the HDAEE group had a significantly lower *IL-6*, *COX-2*, and *mPGES-1* levels compared to the HD group (*p* < 0.05). On days 35 and 42, the levels of EP2 and EP4 expression were significantly higher in the HD group compared to the ND group. Conversely, the levels of EP2 and EP4 were decreased in the HDAEE animals compared to the HD group. The expression levels of prostaglandin E2 receptor 3 (*EP3*) were similar in all treatment groups.

**Table 7:** Effect of AEE on the relative mRNA expression of ileum inflammatory factors in HDS broilers.

Items	ND	HD	NDAEE	HDAEE	SEM	*p* value
IL-1β						
21d	1.00^b^	1.37^a^	1.04^b^	1.07^b^	0.097	<0.01
28d	1.00^b^	1.55^a^	1.10^b^	1.03^b^	0.103	0.02
35d	1.00^b^	1.49^a^	1.06^b^	1.02^b^	0.078	<0.01
42d	1.00^c^	1.59^a^	1.07^c^	1.04^b^	0.074	0.03
IL-6						
21d	1.00	1.07	1.09	1.03	0.081	0.48
28d	1.00^b^	1.32^a^	1.04^b^	1.06^b^	0.106	0.03
35d	1.00^b^	1.45^a^	1.09^b^	1.07^b^	0.061	0.04
42d	1.00^c^	1.53^a^	1.03^c^	1.28^b^	0.082	<0.01
IL-10						
21d	1.00^b^	1.13^a^	1.08^b^	1.06^b^	0.094	0.04
28d	1.00^b^	1.40^a^	1.12^b^	1.23^b^	0.066	<0.01
35d	1.00^b^	1.53^a^	0.75^c^	1.03^b^	0.065	<0.01
42d	1.00^c^	1.62^a^	1.07^c^	1.29^b^	0.071	0.03
TNF-α						
21d	1.00^b^	1.33^a^	1.06^b^	1.14^b^	0.101	0.04
28d	1.00^b^	1.25^a^	1.04^b^	1.18^ab^	0.083	0.02
35d	1.00^bc^	1.43^a^	0.99^c^	1.19^b^	0.063	0.02
42d	1.00^c^	1.58^a^	1.03^c^	1.25^b^	0.082	<0.01
COX-2						
21d	1.00	1.02	1.14	1.03	0.077	0.59
28d	1.00^b^	1.33^a^	1.06^b^	1.06^b^	0.068	0.03
35d	1.00^c^	1.32^a^	1.01^c^	1.16^b^	0.045	<0.01
42d	1.00^c^	1.47^a^	0.99^c^	1.29^b^	0.064	<0.01
mPGES-1						
21d	1.00	1.04	1.19	1.07	0.062	0.63
28d	1.00^bc^	1.26^a^	1.02^c^	1.14^b^	0.049	<0.01
35d	1.00^b^	1.46^a^	1.00^b^	1.16^b^	0.059	0.01
42d	1.00^bc^	1.42^a^	0.98^c^	1.17^b^	0.047	0.03
EP2						
21d	1.00	0.99	1.03	1.06	0.039	0.21
28d	1.00	1.05	1.07	1.03	0.055	0.39
35d	1.00^b^	1.39^a^	1.10^b^	1.11^b^	0.067	0.02
42d	1.00^b^	1.43^a^	1.08^b^	1.22^ab^	0.082	<0.01
EP3						
21d	1.00	1.07	1.03	1.02	0.066	0.35
28d	1.00	0.98	1.00	0.99	0.071	0.43
35d	1.00	1.14	1.07	1.03	0.076	0.37
42d	1.00	1.05	1.01	1.09	0.044	0.56
EP4						
21d	1.00	1.02	1.08	1.04	0.049	0.63
28d	1.00	1.02	0.98	1.02	0.059	0.47
35d	1.00^c^	1.41^a^	1.14^bc^	1.28^ab^	0.072	<0.01
42d	1.00^b^	1.46^a^	1.08^b^	1.25^ab^	0.056	0.02

Means lacking a common superscript in a row are significantly different (*n* = 6, *p* < 0.05).

### 3.6 Effect of AEE on ileum microbiota in HDS broilers

**Figure 2:**
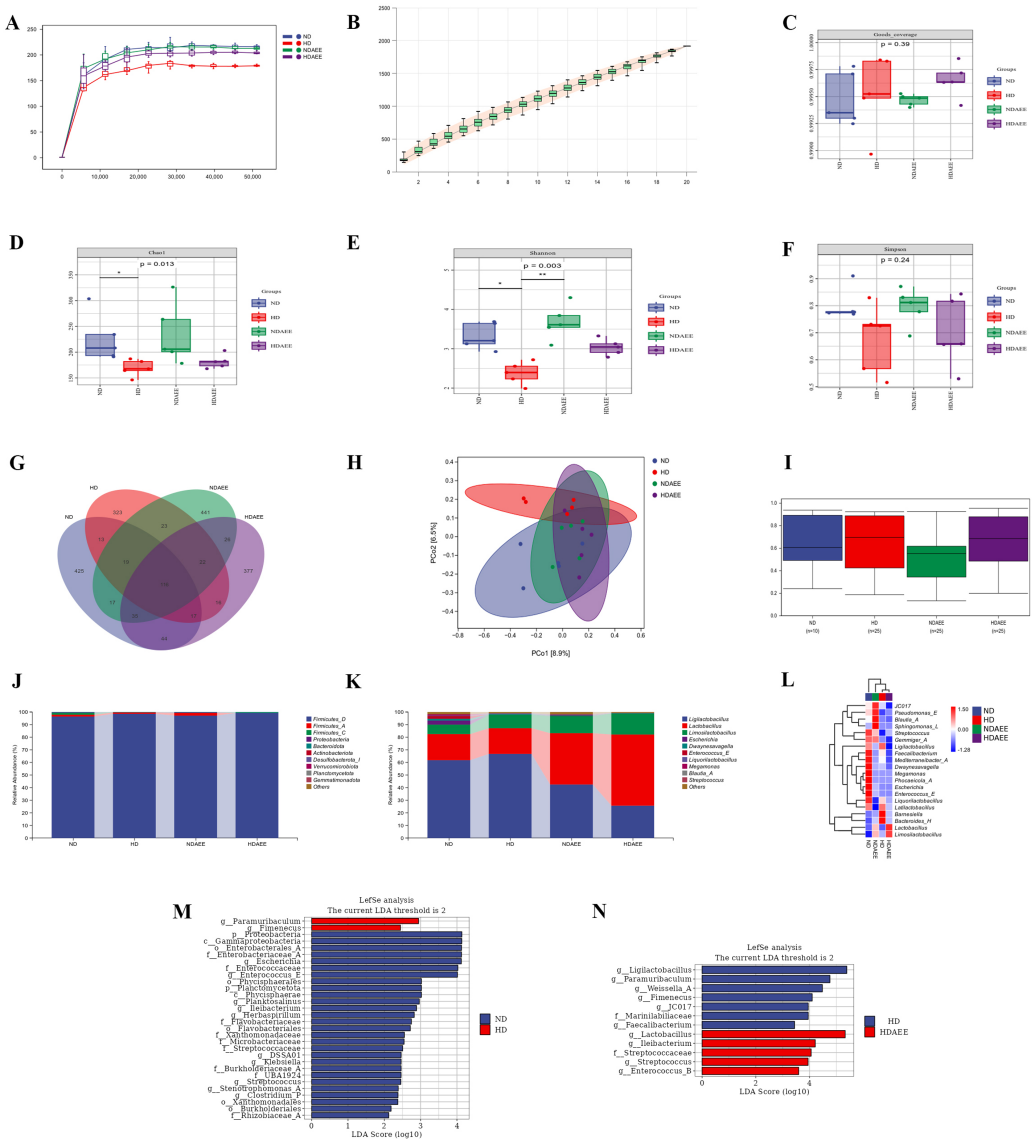
Effect of AEE on ileum microbiota of HD broiler. **(A)** Sparse curve; **(B)** Species accumulation curve; **(C)** Goods coverage index; **(D)** Chao1 index; **(E)** Shannon index; **(F)** Simpson index; **(G)** Microbiota OTU Composition Wayne Diagram; **(H)** Jaccard distance-based principal coordinate analysis (PCOA) of ileum microorganisms; **(I)** Intergroup bacterial community structure analyzed by permanova test; **(J)** phylum-level microbiota composition; **(K)** genus-level microbiota composition; **(L)** Heat map of clustering of the top 20 genus levels in each sample. Differences in ileum microorganisms between ND group and HD group **(M)**, HD group and HDAEE group **(N)** were analyzed by linear discriminant analysis and effect sizes (*n* = 5, *p* < 0.05).

Finally, the influence of AEE on the ileum microbiota community of HD broilers was analyzed. At a sequencing depth of 45,000, the rarefaction curve showed slower growth approaching saturation, indicating that the total number of ileum microbiota species no longer increased significantly with the inclusion of new samples. The good coverage index for each treatment group exceeded 99.93%, indicating that the sequencing depth and sample size were sufficient to represent the majority of species in the samples (Figures 2A–C). In the analysis of ileum microbiota alpha diversity, the Chao1 and Shannon indices were significantly reduced in the HD group compared to the ND group. Compared to the HD group. Chao1 and Shannon indices were increased in the HDAEE group compared to the HD group. No significant differences in the Simpson index were observed between the treatment groups (Figures 2D–F; Supplementary Tables S1–S3). The Venn diagrams revealed the total number of operational taxonomic units (OTUs) in each treatment group: 686 (ND), 549 (HD), 699 (NDAEE), and 653 (HDAEE). A total of 116 OTUs were common across all four groups, while the OTUs for each treatment group were 425 (ND), 323 (HD), 441 (NDAEE), and 377 (HDAEE) (Figure 2G).

To assess the similarity of the microbiota communities between the clades, a Jaccard-based distance matrix downscaling analysis was performed on the ileum microbiota of the four groups. Confidence ellipse distances indicated differences in species composition. Specifically, the ND group was significantly separated from the HD group (*p* = 0.043), but not from the NDAEE group. Moreover, the HDAEE group was separate from the HD group but not from the ND group (Figure 2H). The boxplot analysis of BETA diversity components indicated that the microbiota dispersion and community composition were greater and more fragmented in the HD group compared to the ND group (Figure 2I). Analysis of the ileum microbiota composition revealed *Firmicutes*, *Proteobacteria*, and *Bacteroidota* as the dominant phyla at the phylum level in all four groups. No significant differences in the relative abundance of the dominant phyla were observed between these groups (Figure 2J; Supplementary Table S4). At the genus level, the dominant phyla in the four groups were *Ligilactobacillus*, *Lactobacillus*, *Limosilactobacillus*, and *Escherichia*. Compared to ND, animals in the HD group had significantly higher levels of *Limosilactobacillus* and significantly lower levels of *Escherichia*. While animals in the HDAEE group had significantly reduced *Ligilactobacillus* and increased *Lactobacillus* and *Limosilactobacillus* levels compared to the HD group (*p* < 0.05). Addition of AEE did not significantly affect the HDS-induced reduction in *Escherichia* abundance (Figure 2K; Supplementary Table S5). Cluster and heat map analysis of the 20 most abundant genera at the genus level indicated species composition similarities between the ND and NDAEE groups, and between the HD and HDAEE groups, respectively, suggesting a significant AEE-mediated effect (Figure 2L).

The LEfSe algorithm used to identify the differences in microbiota species composition between treatment groups by using bacteria as biomarkers– revealed higher levels of *Paramuribaculum* and *Fimenecus* and lower levels of *Enterococcaceae*, *Enterococcus*, *Escherichia*, *Ileibacterium* in animals of the HD groups compared to the ND group (Figure 2M). Additionally, animals in the HDAEE group had higher levels of *Lactobacillus*, *Ileibacterium*, *Enterococcus*, *Streptococcaceae*, and *Streptococcus* and lower levels of *Ligilactobacillus*, *Paramuribaculum*, *Weissella*, *Fimenecus*, and *JC017* compared to the HD group (Figure 2N).

## 4 Discussion

Despite the economic benefits of large-scale intensive farming, HDS has a negative impact on animal welfare by affecting animal behavior, nutritional metabolism, physiological activities, and neurological development. Consequently, this prolonged exposure to unfavorable growing conditions results in chronic stress and impaired animal health (44). In fact, stress-induced activation of physiological stress pathways in broilers resulting in reduced feed intake and body weight gain, abnormal serum and tissue biochemical indices, impaired broiler performance, and deleterious inflammatory responses were previously shown (45, 46). Moreover, stocking densities above 18 birds/m2 consistently resulted in a reduced production performance exemplified by decreased broiler feed intake and average daily weight gain and increased FCR (47, 48).

The current study was divided into two main phases: the starter-grower phase (1–21 days) and the finisher phase (22–42 days). During the starter-grower phase, no significant difference in production performance between treatment groups was observed. During the finisher phase, HDS reduced the production performance of broilers by decreasing ADFI and ADG. Furthermore, animals in the HDAEE group had increased ADG and decreased FCR compared with the HD group, while no significant effect on the ADFI was observed. These observations suggest AEE supplementation in feed can improve production performance by increasing ADG under large-scale intensive farming conditions. Large-scale intensive farming conditions expose broilers to a higher environmental temperature and increased atmospheric concentrations of toxic and hazardous gasses. In fact, increased ROS production in broilers disrupts the oxidative and antioxidant balance in the body (47, 48).

The interplay between SOD, CAT, and T-AOC is crucial to maintain the oxidative-antioxidant balance and to protect cellular health. Also, the MDA level –a marker of intracellular oxidative lipid damage– is directly related to oxidative stress (49). In the current study, HDS resulted in decreased ileum SOD and CAT activities, decreased T-AOC activity, and increased MDA content, indicating density stress-induced oxidative damage in the ileum as well as impaired nutrient absorption and metabolism. Addition of AEE to the diet of animals kept under large-scale intensive farming conditions attenuated the HDS-induced decrease in SOD, CAT, and T-AOC activities, as well as the increase in MDA level. These findings suggest that, by improving the body’s antioxidant properties, AEE preserves normal intestinal physiology.

Next, to serving as the primary site of nutrient absorption, the intestinal tract acts as an essential line of defense against pathogenic microorganisms and toxins. However, for effective nutrient absorption and defense against toxins and pathogens, intact intestinal morphology and physical barrier integrity are critical (50, 51). For example, nutrient absorption efficiency correlates with villi length and width (26, 52), while crypt depth correlates with cell regeneration and renewal, the maintenance of intestinal barrier function and regulation of the immune response, as well as nutrient transport (53, 54). At 21 and 28 days of age, elevated levels of stress resulted in a substantial reduction in the height of ileum villi, accompanied by a certain degree of villus morphology damage. This was concomitant with a decline in crypt depth. However, the addition of AEE to the diet was efficacious in ameliorating the damage to villus morphology and the decrease in villus height. At 35 and 42 days of age, as the body weight of the broilers increased, the damage to the intestinal villi was exacerbated by elevated levels of high-density stress. The addition of AEE to the diets was not efficacious in alleviating the damage to villus morphology, although it could significantly mitigate the decrease in villus height. The findings of the present study indicate that AEE has the capacity to mitigate the damage to the ileum caused by high-density stress to a certain extent. However, this mitigation is progressively diminished as the body weight of the broiler increases. Given that intestinal barrier damage correlates with production performance, it is likely that HDS-induced declines in production performance are mediated by intestinal barrier damage. Addition of AEE to the diet attenuated the HDS-induced VH decrease and CD increase to some extent and preserved production performance and intestinal barrier integrity. These findings further suggest that AEE might improve HDS-mediated declines in production performance by preserving the intestinal barrier function.

The tight junction proteins *occludin*, *claudin*, and *ZO-1* are critical to maintain intestinal barrier integrity and preservation of nutrient absorption and metabolism. Also, these proteins are involved in the transmission and activation of immune cells, what eventually could result in the development of inflammatory bowel disease (55, 56). Previous research indicated that HDS hinders normal intestinal development resulting in intestinal barrier damage through diminished *occludin*, *claudin-1* and *ZO-1* expression in the epithelial lining of the small intestine (23, 57, 58). Consistent with these findings, the current study evidenced a significant HDS-mediated decrease in ileum *claudin-2* and *ZO-2*, which was prevented upon supplementing the feed with AEE. These findings suggest AEE supports proper intestinal barrier development and maintenance by preserving tight junction protein expression. Intestinal barrier damage leads to increased intestinal permeability thereby undermining the intestinal tract resistance to external pathogenic microorganisms (59–61). Subsequent entry of pathogenic microorganisms into the body causes cell damage, which in turn could lead to inflammatory bowel disease (62, 63). Inflammatory signaling pathway activation results in *IL-1β*, *IL-6*, and *TNF-α* upregulation in the intestine. Chronic excess of inflammatory cytokines disrupts the integrity of intestinal epithelial cells, increases intestinal permeability, and ultimately leads to a broader inflammatory response and immune disorders (60).

The current study evidenced an HDS-mediated upregulation of *IL-1β*, *TNF-α*, and *COX-2* as well as an increase of the *COX-2/PGE2* pathway downstream receptors *EP2* and *EP4*, which was attenuated by AEE. Among them, HDS resulted in elevated expression of *EP2* and *EP4* genes in broilers, which may lead to activation of the *NF-κB* signaling pathway, which in turn produces an inflammatory response. The present findings suggest that HDS induces an inflammatory response mediated by the *COX-2-mPGES-1* signaling pathway, leading to a compromised intestinal barrier, impaired nutrient absorption, and decreased productive performance. The addition of AEE to the diet has been shown to alleviate the inflammatory response by inhibiting the expression of genes related to the COX-2-mPGES-1 signaling pathway, thus restoring the normal physiological function of broilers and improving their performance.

While gut microbiota, encompassing fungi and bacteria, are crucial for digestion, nutrient absorption, neuroendocrine balance, energy metabolism, and immune regulation, certain microorganisms can pose risks (64, 65). Previous studies highlighted the importance of highly biodiverse, stable, and homogeneous microbiota composition to maintain the intestinal barrier. In fact, disturbance of the intestinal microbiota by external pathogenic microorganisms or harsh environments results in intestinal barrier damage, intestinal inflammation, as well as impaired nutrient absorption and metabolism (66, 67). HDS was previously shown to disturb the caecum microbiota of broilers (68), which is consistent with the findings of the current study showing disrupted microbiota homeostasis in the ileum. Specifically, compared to the ND group, the HD group had significantly reduced Chao1 and Shannon indices (*p* < 0.05), while the beta diversity analysis showed a significant separation of species composition (*p* < 0.05). Consistent with previous experiments in rat (42), adding AEE to the diet mitigated the HDS-induced decline in alpha diversity and reduced the difference in beta diversity between high and low densities. These results indicate that AEE alleviates microbiota diversity imbalances caused by external stimuli, thereby facilitating a species-rich and stable microbiota community.

In poultry, the intestinal microbiota community—comprising a complex micro-ecosystem of probiotic and potentially pathogenic microorganisms—contributes to a normal intestinal physiology (69). Of these, the phyla *Bacteroidetes* and *Firmicutes* are the most dominant phyla in the poultry intestine, while *Proteobacteria* particularly abundant in the ileum (70). In the current study, the combined relative abundance of the *Bacteroidetes* and *Firmicutes* phyla exceeded 98%. While no significant differences in *Bacteroidetes* phyla were observed between the groups, the relative abundance of *Firmicutes* phylum was lower in the HD group. Furthermore, compared to the ND group, animals in the HD group had a lower relative abundance of *Bacteroidota* phylum. Previously, a positive correlation between the *Bacteroidetes* to *Firmicutes* phyla ratio and growth and development rates was shown (71). Consistent with these findings, the current study reported an increased ratio of *Bacteroidetes* to *Firmicutes* phyla, as well as a decreased production performance. At the genus level, an HDS-induced reduction in the relative abundance of *Lactobacillus*, *Dwaynesavagella*, and *Megamonas* was shown. Previously, the ability of *Lactobacillus* and *Megamonas* to prevent pathogenic microorganisms from colonizing the intestine through intestinal pH-regulation was reported (72, 73). Moreover, *Megamonas*—including some subspecies—can ferment acetic and butyric acids, which are critical for maintaining a healthy intestinal barrier and modulation of the immune response. Also, *Dwaynesavagella* is strongly associated with the segmented filamentous bacterium SFB in the ileum, which is crucial for building the intestinal barrier through stimulating intestinal mucosal epithelial cell proliferation (74). Consequently, an HDS-mediated decline in the relative abundance of beneficial bacteria, such as *Lactobacillus* and *Megamonas*, might underlie impairment of the ileum-intestinal barrier and subsequent development of inflammatory bowel disease. Addition of AEE to the diets of animals reared under high-density growth conditions significantly increased the relative abundance of *Lactobacillus* but decreased the relative abundance of *Ligilactobacillus*. The fact that this finding correlated with decreased levels of inflammatory factors in the current study suggests that AEE might alleviate intestinal inflammation through establishment of a physiological normal intestinal microbiota (75).

Previously, AEE-mediated reversal of hyperlipidemia-induced decline in microbiota diversity in a high-fat diet rat model was shown. As AEE was shown to enhance the relative abundance of *Lactobacillus*, it suggests that AEE-mediated improvement in the intestinal microbiota diversity may underlie its ability to attenuate the negative effects of hyperlipidemia (76). While the current study confirms these previous observations, an AEE-mediated increase in *Limosilactobacillus*—a new branch of Lactobacillus in the Greengens2 database—was shown. Interestingly, *Limosilactobacillus* is involved in regulating nutrient absorption and can improve intestinal immune function (77). In the current study, LEfSe analysis indicated an AEE-mediated increase in mainly *Lactobacillus* and *Ileocecum* and, to a lesser extent, *Streptococcus* and *Enterococcus* in broilers raised under high density growing conditions. These observations suggest that AEE may alleviate the negative effects of HDS by influencing the structural composition of the gut microbiota.

**Figure 3:**
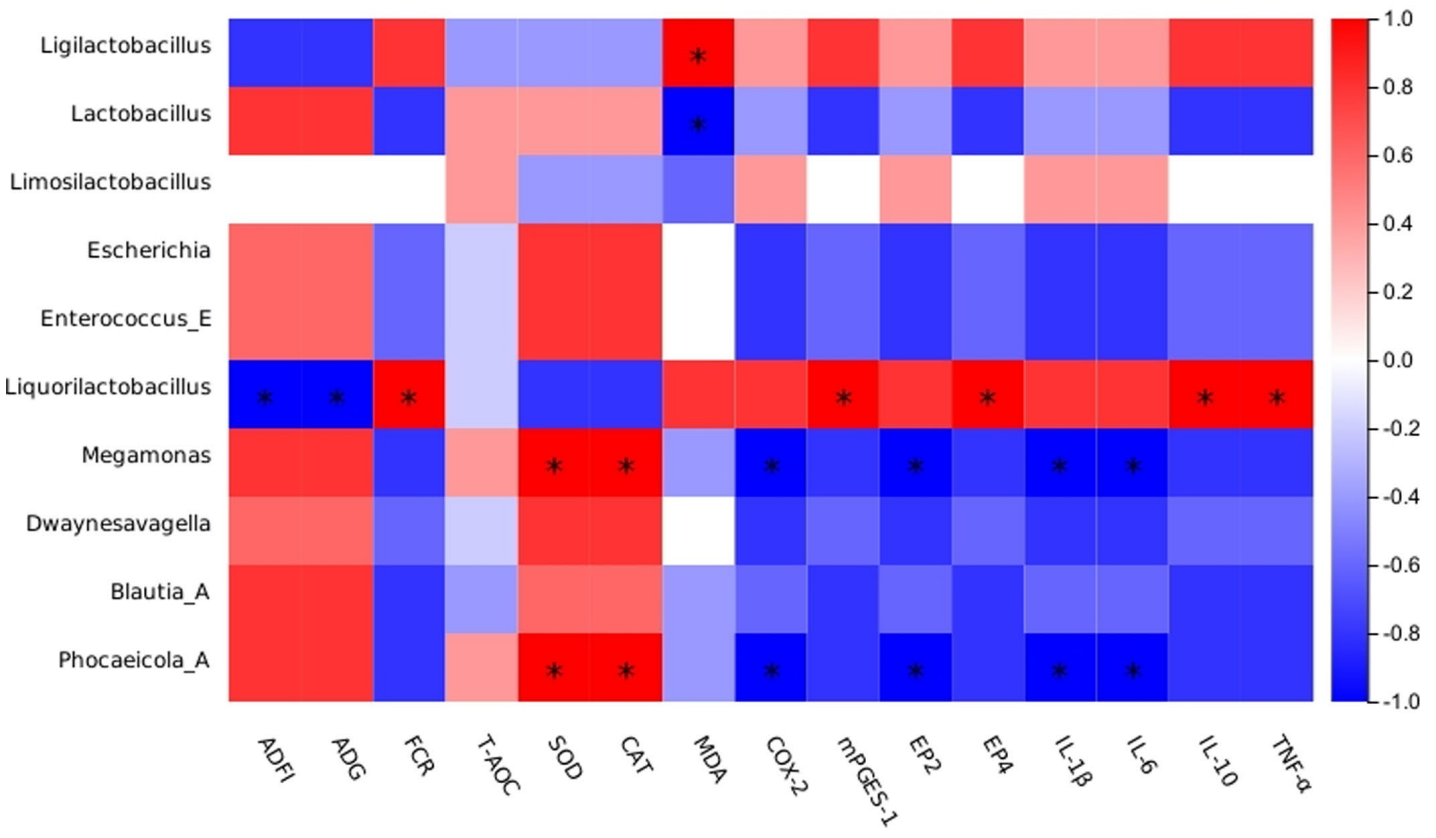
Spearman’s correlation analysis between the abundances of intestinal microbiota and phenotypes. Red represents a positive correlation, and blue represents a negative correlation. Significant correlations are noted by * and represent 0.01 < *p* ≤ 0.05.

The current study evidenced a strong correlation between the abundance of *Lactobacillus* and *Megamonas* in the ileum microbiota and production performance (Figure 3). Also, a significant negative correlation between *Lactobacillus* and MDA was shown. Previously, *Lactobacillus* was shown to reduce intestinal colonization by pathogenic microorganisms while maintaining the intestinal barrier integrity (78). Consistent with previous findings, *Megamonas* abundance showed a significant positive correlation with SOD and CAT levels, as well as a significant negative correlation with the inflammatory factor *COX-2*. However, the current results only show a certain correlation between them, and the correlation between them and the mechanism of influence still need to be further explored.

Stress accompanies almost every process of animal production. Traditional anti-stress supplements, such as vitamins, can alleviate heat stress and reduce oxidative damage, but they are prone to degradation and failure in high temperature or humid environments, and the dosage is difficult to control, which is easy to cause damage to the organism. The effective dose of plant extracts is difficult to standardize, and the palatability of some extracts is poor, which affects the feed intake and thus the production performance. At the same time, the high extraction cost of plant extracts is also a major problem in their actual production. Other organic acids and probiotics, in addition to poor palatability, affect production performance, and are easily inactivated during high-temperature pelleting and storage, affecting their original functions. In addition, the above anti-stress supplements have a single target and often need to be used in combination, AEE is a colorless and tasteless crystal. It is also physically and chemically stable, making it easy to transport and store. Meanwhile, AEE has been proved to be non-toxic, low residual and has a wide range of targets. Therefore, AEE is expected to be a new type of anti-stress supplement with high efficiency, low residue, low toxicity and a wide range of targets.

## 5 Conclusion

**Figure 4:**
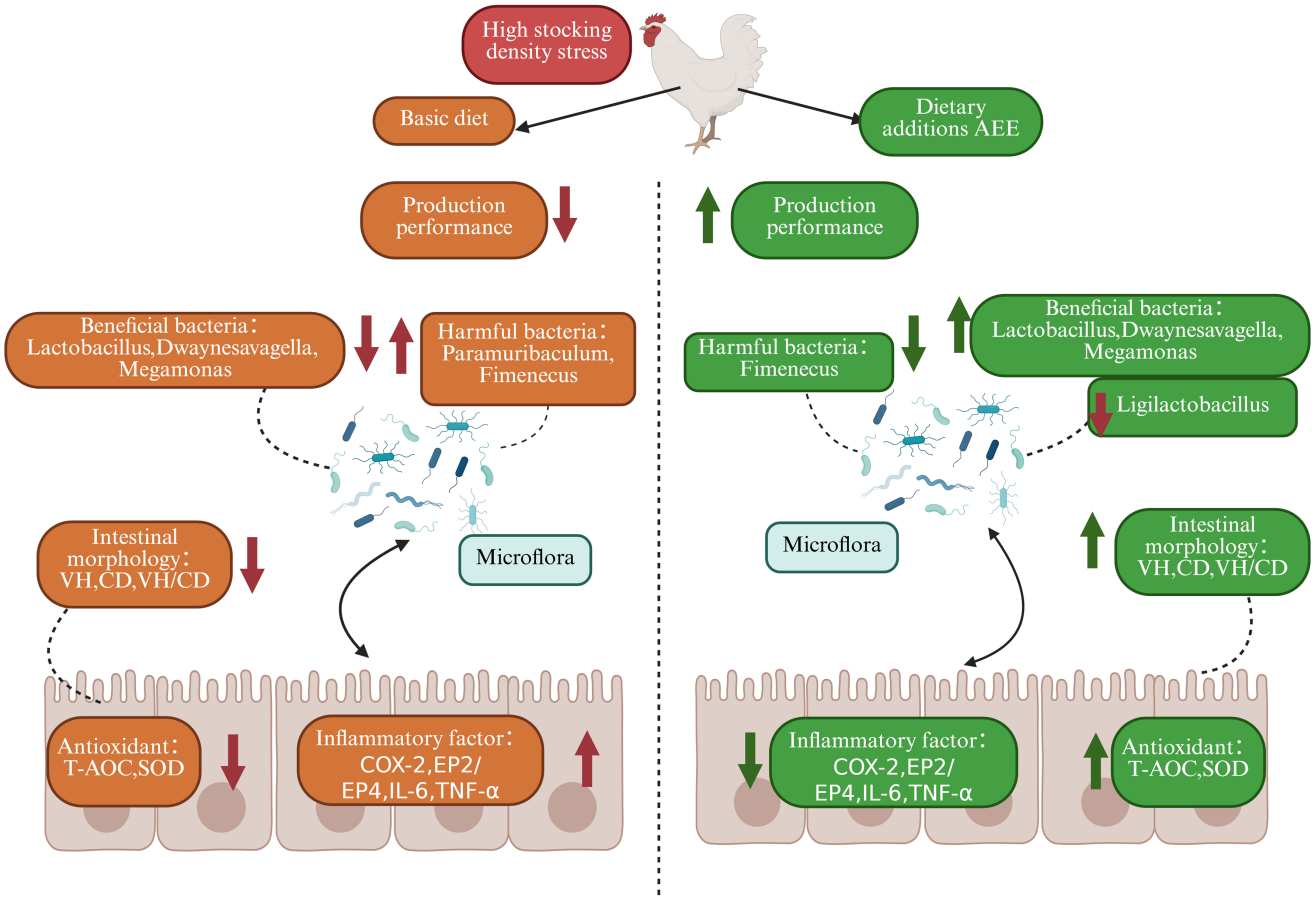
Effect of AEE on production performance, intestinal barrier, inflammation and microbial diversity in HDS broilers.

This study evidenced that HDS in broilers impaired growth performance and ileum intestinal barrier function, induced intestinal inflammation, and reduced intestinal antioxidant properties. Moreover, HDS-mediated deterioration of the ileum microbiota was shown as exemplified by significantly lower levels of *Lactobacillus* and *Megamonas*. Addition of AEE to broiler diets may alleviate to some extent the HDS-induced decline in growth performance, impairment of gut barrier function, and decrease in gut microbiota diversity. It is evident that AEE exerts a multifaceted influence on promoting growth, encompassing several interconnected mechanisms. These include the preservation of intestinal barrier integrity and the stability of microbiota, the attenuation of inflammation, and the restoration of intestinal antioxidant capacity. The inhibition of relevant genes along the *COX-2-mPGES1* signaling pathway appears to be a pivotal mechanism through which these effects are achieved (Figure 4).

## Data Availability

The datasets presented in this study can be found in online repositories. The names of the repository/repositories and accession number(s) can be found in the article/Supplementary material.

## Footnotes

^1^ https://docs.qiime2.org/2019.4/tutorials/

^2^ https://www.genescloud.cn
