# Supplementary material for: Effect of dietary supplementation with aspirin eugenol ester on performance and ileum health in broilers under high stocking density stress conditions
Source: Front Vet Sci. 2025 Jul 7;12:1638245. doi: 10.3389/fvets.2025.1638245 (PMC12277146; doi:10.3389/fvets.2025.1638245)
Supplement: Supplementary file 1 [file Table_1.docx]

Supplementary Material

**Table S1.** Chao1 index test table

| Group | ND | HD | NDAEE |
| --- | --- | --- | --- |
| *HD* | 0.038 | NaN | NaN |
| *NDAEE* | 0.87 | 0.051 | NaN |
| *HDAEE* | 0.22 | 0.85 | 0.23 |

ND, normal stocking density fed basal diet; HD, high stocking density fed basal diet; ND+AEE normal stocking density fed basal diet supplemented with 0.01% AEE; HD+AEE high stocking density group fed basal diet supplemented with 0.01% AEE. Chao1 index: Chao1 richness estimator

**Table S2.** Shannon index test table

| Group | ND | HD | NDAEE |
| --- | --- | --- | --- |
| *HD* | 0.023 | NaN | NaN |
| *NDAEE* | 0.52 | 0.0031 | NaN |
| *HDAEE* | 0.52 | 0.31 | 0.31 |

ND, normal stocking density fed basal diet; HD, high stocking density fed basal diet; ND+AEE normal stocking density fed basal diet supplemented with 0.01% AEE; HD+AEE high stocking density group fed basal diet supplemented with 0.01% AEE. Shannon index: Shannon diversity index

**Table S3.** Simpson index test table

| Group | ND | HD | NDAEE |
| --- | --- | --- | --- |
| *HD* | 0.67 | NaN | NaN |
| *NDAEE* | 0.88 | 0.47 | NaN |
| *HDAEE* | 0.93 | 0.82 | 0.8 |

ND, normal stocking density fed basal diet; HD, high stocking density fed basal diet; ND+AEE normal stocking density fed basal diet supplemented with 0.01% AEE; HD+AEE high stocking density group fed basal diet supplemented with 0.01% AEE. Simpson index: Simpson's diversity index

**Table S4.** Species composition at the ileum microbiota level.

| Microorganisms | ND | HD | NDAEE | HDAEE | SEM | P-value |
| --- | --- | --- | --- | --- | --- | --- |
| *Firmicutes_D* | 96.36% | 98.72% | 97.16% | 99.24% | 0.186 | 0.212 |
| *Firmicutes_A* | 1.36% | 0.83% | 2.20% | 0.21% | 0.01 | 0.102 |
| *Firmicutes_C* | 1.53% | 0.04% | 0.07% | 0.07% | 0.009 | 0.166 |
| *Proteobacteria* | 0.27%^ab^ | 0.17%^b^ | 0.33%^a^ | 0.22%^ab^ | 0.001 | 0.024 |
| *Bacteroidota* | 0.36% | 0.14% | 0.14% | 0.14% | 0.001 | 0.084 |
| *Desulfobacterota_I* | 0.05% | 0.01% | 0.01% | 0.02% | 0.0002 | 0.14 |
| *Verrucomicrobiota* | 0.01% | 0.02% | 0.01% | 0.02% | 0.00005 | 0.38 |

Means lacking a common superscript in a row are significantly different (n = 5, P < 0.05).

**Table S5.** Genus-level species composition of ileum microorganisms.

| Microorganisms | ND | HD | NDAEE | HDAEE | SEM | P-value |
| --- | --- | --- | --- | --- | --- | --- |
| *Ligilactobacillus* | 61.89%^a^ | 66.85%^a^ | 42.46%^b^ | 25.50%^b^ | 0.194 | 0.043 |
| *Lactobacillus* | 20.66%^b^ | 20.36%^b^ | 40.72%^a^ | 56.71%^a^ | 0.167 | 0.041 |
| *Limosilactobacillus* | 7.52% | 10.66% | 13.71% | 16.55% | 0.062 | 0.199 |
| *Escherichia* | 3.01%^a^ | 0.06%^b^ | 0.40%^b^ | 0.04%^b^ | 0.004 | 0.003 |
| *Dwaynesavagella* | 1.71%^a^ | 0.38%^c^ | 1.31%^b^ | 0.31%^c^ | 0.011 | 0.041 |
| *Enterococcus_E* | 1.81%^a^ | 0.03%^c^ | 0.21%^b^ | 0.01%^c^ | 0.008 | 0.001 |
| *Liquorilactobacillus* | 0.63% | 0.46% | 0.24% | 0.35% | 0.002 | 0.162 |
| *Megamonas* | 0.59%^a^ | 0.04%^b^ | 0.44%^a^ | 0.04%^b^ | 0.006 | 0.036 |
| *Blautia_A* | 0.07% | 0.03% | 0.15% | 0.02% | 0.001 | 0.228 |
| *Streptococcus* | 0.06% | 0.03% | 0.05% | 0.04% | 0.001 | 0.066 |

Means lacking a common superscript in a row are significantly different (n = 5, P < 0.05).
